# The Tubulin Code and Tubulin-Modifying Enzymes in Autophagy and Cancer

**DOI:** 10.3390/cancers14010006

**Published:** 2021-12-21

**Authors:** Daniela Trisciuoglio, Francesca Degrassi

**Affiliations:** IBPM Institute of Molecular Biology and Pathology, CNR Consiglio Nazionale delle Ricerche, c/o Department of Biology and Biotechnology “Charles Darwin”, Sapienza University of Rome, 00185 Roma, Italy

**Keywords:** microtubules, tubulin post-translational modifications, tubulin-modifying enzymes, acetylation, tyrosination, cancer, autophagy

## Abstract

**Simple Summary:**

Microtubules are tubulin polymers that constitute the structure of eukaryotic cells. They control different cell functions that are often deregulated in cancer, such as cell shape, cell motility and the intracellular movement of organelles. Here, we focus on the crucial role of tubulin modifications in determining different cancer characteristics, including metastatic cell migration and therapy resistance. We also discuss the influence of microtubule modifications on the autophagic process—the cellular degradation pathway that influences cancer growth. We discuss findings showing that inducing microtubule modifications can be used as a means to kill cancer cells by inhibiting autophagy.

**Abstract:**

Microtubules are key components of the cytoskeleton of eukaryotic cells. Microtubule dynamic instability together with the “tubulin code” generated by the choice of different α- and β- tubulin isoforms and tubulin post-translational modifications have essential roles in the control of a variety of cellular processes, such as cell shape, cell motility, and intracellular trafficking, that are deregulated in cancer. In this review, we will discuss available evidence that highlights the crucial role of the tubulin code in determining different cancer phenotypes, including metastatic cell migration, drug resistance, and tumor vascularization, and the influence of modulating tubulin-modifying enzymes on cancer cell survival and aggressiveness. We will also discuss the role of post-translationally modified microtubules in autophagy—the lysosomal-mediated cellular degradation pathway—that exerts a dual role in many cancer types, either promoting or suppressing cancer growth. We will give particular emphasis to the role of tubulin post-translational modifications and their regulating enzymes in controlling the different stages of the autophagic process in cancer cells, and consider how the experimental modulation of tubulin-modifying enzymes influences the autophagic process in cancer cells and impacts on cancer cell survival and thereby represents a new and fruitful avenue in cancer therapy.

## 1. Introduction

### 1.1. Microtubule Dynamic Instability

Microtubules (MTs) are hollow cylinders of approximately 25 nm formed by the polymerization of α-/β-tubulin dimers. MT polymers are intrinsically polar with a faster addition and release of tubulin dimers at the plus end and a slower dynamics at the more stable minus end.

In interphase cells, MTs are key components of the filamentous cytoskeletal network, acting in concert with actin filaments and intermediate filaments. In most cell types, MTs regulate the spatial organization of the cell, contributing to cell shape and cell motility, and control intracellular spatial functions, such as organelle positioning and intracellular transport. MTs have a fundamental role in intracellular trafficking, allowing the movement of signaling molecules or organelles, such as the endoplasmic reticulum, the Golgi complex, mitochondria, and autophagic vesicles. In this regard, MTs function as preassembled tracks on which kinesins (plus-end-directed) or cytoplasmic dynein (minus-end-directed) motor proteins transport cargoes by exerting pulling and pushing forces on dynamic MTs or by sliding interdigitating MTs (for a review see [1]).

In dividing cells, MTs control the process of mitosis at different stages of its progression. At mitotic entry, the MT network extensively reorganizes to form the mitotic spindle, a fusiform structure of two radial arrays of short, dynamic MTs that govern chromosome segregation. At cytokinesis, cytoplasmic division intervenes at the residual MT structure called midbody. At the heart of these different functions lies the “dynamic instability” of MTs, the filament property for which individual MTs undergo continuous cycles of rapid depolymerization (catastrophe) and polymerization (rescue) at their plus end [2]. Dynamic instability continuously remodels the MT network, allowing individual MTs to explore the intracellular space, a property that is fundamental during mitosis when MTs grow and shrink rapidly until they bind a chromosome at kinetochore [2]. This binding restrains MT dynamics, allowing a robust attachment of chromosomes to the mitotic spindle, a prerequisite for correct chromosome segregation. Indeed, control of MT dynamics at mitosis has been shown to be fundamental in faithful chromosome segregation, so that altered MT dynamics can lead to chromosome segregation errors and chromosome instability, a key feature of cancer cells. A plethora of studies in the last 20 years have delineated the connection between cancer, chromosome instability, and control of MT dynamics, and these factors are discussed in several excellent reviews to which the reader can refer [3,4,5]. Importantly, enhanced tubulin dynamics at mitosis is also the molecular target for numerous so-called anti-tubulin agents that have been shown to interact with several sites on α- or β-tubulin and have been successfully used as chemotherapeutic drugs to cause mitotic arrest and cell death of cancer cells [6,7,8,9].

### 1.2. The α-/β-Tubulin Code

The tubulin superfamily in humans comprises five families: alpha-(α), beta-(β), gamma-(γ), delta-(δ), and epsilon-(ε) tubulin. Nine α-tubulin (TubA), ten β-tubulin (TubB), two γ-tubulin (TubG), one δ-tubulin (TubD), and one ε-tubulin (TubE) isoforms have been identified. Tubulin isoforms derive from different genes that are located on different chromosomes, and their aberrant expression or mutation is associated with several human pathologies [10,11]. TubA and TubB isoforms are the main components of MTs, and they share a high degree of homology at the N-terminal and intermediate domain, while they differ significantly in the length of the C-terminal tail, the region that harbors interaction sites for MT-associated proteins (MAPs) and MT molecular motors [12,13,14,15].

TubG is the main component of multiprotein complexes that are found at MT organ-izing centers, such as centrosomes in most animal cells. TubG-containing complexes nucleate MTs both at MT organizing centers and at other intracellular sites, such as the Golgi apparatus and preexisting MTs [16,17,18]. TubD and TubE are also centrosomal proteins, but they display specific localization patterns: TubD is associated with centrioles, whereas TubE localizes to the pericentriolar material with a cell cycle-specific localization pattern [19].

In the last years, the idea that different TubA and TubB gene products, together with a variety of tubulin post-translational modifications (PTMs), generate a code, named the “tubulin code”, has attracted a great deal of interest [20]. Hence, individual MTs, harboring a specific tubulin code, have been shown to have different properties or architectures between cell types or even within a single cell due to their peculiar ability to interact with MAPs or MT molecular motors [21,22].

TubA and TubB isoforms are for the most part ubiquitously expressed but some of them have cell- or tissue-specific expression. For example, TubB1 is expressed only in platelets and megakaryocytes [15], whereas TubB3 is physiologically expressed in cells of neuronal origins [12,13,14] and TubB4 in the axonemes of cilia and flagella [23]. The tubulin isotype composition of MTs has a clear impact on MT dynamics. In 1994, Panda and coauthors showed that MTs composed of TubA and TubB3 isotypes exhibit unique polymerization dynamics, being more dynamic than MTs formed of TubA and TubB2 or TubB4 isotypes [24]. A more recent study, comparing the dynamics of neuronal MTs and MTs obtained from human embryonic kidney cells, has shown that the latter MTs are more stable and grow faster than neuronal MTs [25]. Overall, these studies confirmed the role of tubulin isoform composition on MT dynamics.

Tubulin PTMs are a key player in the tubulin code, and they have been shown to exert a clear impact on different MT functions. In the last few years, the discovery of tubulin-modifying enzymes has allowed researchers to decipher the role of tubulin PTMs in MT dynamics (Table 1) and to unveil the mechanisms by which specific PTMs regulate MT function in different cell contexts (for a comprehensive review see [26]). PTMs relevant to cancer occur abundantly and reversibly on tubulin and are mostly localized at the C-terminal tail of the protein; some PTMs, including acetylation, detyrosination, and polyglutamylation, are specifically associated with polymerized tubulin, while others, like tyrosination, occur only on soluble tubulin; polyamination intervenes both on soluble and polymerized tubulin.

Tubulin acetylation refers to the transfer of the acetyl group from acetyl-coenzyme A (CoA) at the Lysine-40 residue (K40) of TubA, which is exposed in the lumen of MTs. In normal cells, acetylated TubA is associated with long-lived stable MTs within centrioles, primary cilia, and flagella [27]. To date, the relationship between tubulin acetylation and MT stability is not fully understood. The predominant idea was that only stable MTs may be acetylated, based on experiments showing that drug-induced MT stabilization determines an accumulation of acetylated MTs [28] or on studies linking decreased tubulin acetylation with reduced MT stability [29]. More recent studies have proved that acetylation reduces the rigidity of MTs, making them more resistant to mechanical breakage and disassembly [30,31]. The alpha-tubulin N-acetyltransferase 1 (ATAT1 or MEC17) has been identified as being responsible for K40 acetylation on TubA in mammalian cells [32], whereas the reverse reaction is catalyzed by the HDAC6 and/or sirtuin type 2 (SIRT2) deacetylases [29,33]. However, other acetyltransferases, such as GCN5, can also acetylate α-tubulin in different cell contexts [34]. More recently, another reaction occurring at Lysine-252 (K252) of TubB, favoring MT depolymerization, has also been described [35]. The acetyltransferase SAN catalyzes K252 acetylation of TubB, whereas the enzyme catalyzing the reverse reaction has not been defined yet [35]. Functionally, tubulin acetylation has been linked to the recruitment of specific motor proteins to MTs and to intracellular trafficking [36,37]. Consequently, tubulin acetylation plays an important role in several cellular activities, including cell polarity, cell migration, vesicle transport, and cell development. Indeed, abnormal tubulin acetylation levels have been linked to a number of different diseases, including ciliopathies, neurodegenerative disorders, and cancer (for a review see [38]). Detyrosination/tyrosination of TubA also occurs in the C-terminal tail and the cycling of this modification on tubulin is closely connected to MT dynamics: newly polymerized MTs are mostly tyrosinated, while long-lived MTs are typically detyrosinated, so that detyrosination has long been considered a marker of MT longevity [39]. The tyrosination state of MTs can also modify the binding affinity for motor proteins: some kinesins bind preferentially detyrosinated MTs, while cytoplasmic dynein interacts more efficiently with tyrosinated MTs [40]. Consequently, specific cargo transport has been found to use different MT subsets, with important implications in cell division, neuronal, and cardiac physiology [41,42,43]. The tubulin terminal tyrosine is removed by cytosolic carboxypeptidases (CCPs) to expose a glutamate, whereas the reverse reaction is catalyzed by tubulin–tyrosine ligase (TTL). Two redundant CCPs, vasohibin-1 (VASH-1) and vasohibin-2 (VASH-2), have been found to detyrosinate MTs in a complex with a chaperone-like small vasohibin-binding peptide (SVBP) [44,45]. The detyrosination/tyrosination cycle is completed by TTL, which binds only tubulin dimers and adds a tyrosine residue to detyrosinated tubulin [46].

Finally, glutamate side chains of different lengths can be added to the C-terminal tail of both TubA and TubB on glutamate residues. This reaction, named mono- or polyglutamylation, adds one or multiple glutamic acids to a γ-carboxyl group of a glutamate residue of tubulin and is catalyzed by several TTL-like (TTLL) mono- or polyglutamylases, a large family of proteins harboring a TTL homology domain. The reverse reaction is carried out by a deglutamylase from the CCP family. Tubulin polyglutamylation, preferentially occurring in cilia MTs, regulates multiple interactions between MTs and their associated proteins, such as MAPs and molecular motors. For instance, the activity of several molecular motors, including kinesin-1 and kinesin-2, can be regulated by tubulin isotypes and various degrees of MT polyglutamylation [37].

Overall, the combination of tubulin PTMs and isotypes may generate a subpopulation of MTs with specific dynamic properties that influence different cellular effectors, such as MAPs or motor proteins. However, work is still needed to determine the impact of less well characterized tubulin PTMs (e.g., methylation, polyamination) on MT structure in diverse cell types.

## 2. The Tubulin Code and Its Associated Enzymes in Cancer

In cancer cells, alterations in MT dynamics, often associated with cancer-specific tubulin isotypes and tubulin PTMs, have been shown to be involved in metastatic cell migration, drug resistance, and tumor vascularization [5,38]. Accordingly, anti-tubulin agents have been proved to have anti-angiogenic and vascular-disrupting properties, as well as effects on cellular migration and intracellular trafficking [47]. However, the relevance of these effects to the anti-tumor activity of anti-tubulin drugs has been overlocked in the past due to the efficacy of their mitotic action.

In several cancer cell contexts, emerging studies highlight a role for tubulin isotypes in influencing MT behavior and function in the metastatic ability and chemotherapy resistance of cancer cells (Table 2). Indeed, a differential TubA1 isotype expression has been found in various types of cancer and correlates with poor outcome/prognosis as well as with resistance to therapy [48,49,50,51,52]. TubA1B expression was found to be upregulated in hepatocellular carcinoma (HCC) tumor tissues and correlated with poor overall survival and resistance to paclitaxel in HCC patients [48]. Recently, it was demonstrated that glioma tissues have higher TubA1C expression than normal brain tissues and that high TubA1C levels are an indicator of worse prognoses in glioma patients, thereby suggesting that TubA1C may be a therapeutic biomarker for gliomas [49,52].

The frequency and impact of TubB isoforms and mutations in cancer is still not fully understood. TubB1 is the most common isotype in human lung cancer and breast cancer cell lines [53]. TubB1 expression is correlated with taxane resistance in breast cancer [54].

Class 3 of TubB is the prominent isoform linked to neoplastic disease and has been identified as a biomarker for resistance to MT-targeting chemotherapeutics in breast and other types of solid cancer [55]. TubB3 overexpression has been linked to aggressive tumor features, genetic instability, and poor prognosis in urinary bladder cancer and clear cell renal cell carcinoma [56,57]. Aberrant expression of TubB3, TubB2, TubB4A, TubB4B, and TubB5 isotypes has been detected in several tumor types and is also associated with resistance to tubulin-binding agents, such as taxanes and vinca alkaloids, in different cancer cell types [54,58,59,60,61,62,63,64,65,66]; this may be due to the specific properties of tubulin isoforms which could alter the sensitivity of MTs to this class of drugs.

Among tubulin PTMs, tubulin acetylation was first described 20 years ago and has recently attracted growing interest in the field of cancer research. Many studies have suggested alterations in tubulin acetylation as potential prognostic biomarkers in different cancers, including head and neck, breast, and pancreatic cancer [67,68]. Boggs and collaborators have linked acetylated tubulin levels to the metastatic process in breast cancer, suggesting a relationship between high levels of acetylation and metastatic behavior of basal-like breast cancers [68]. More recently, acetylated tubulin levels have been associated with paclitaxel sensitivity in lung cancer. Indeed, tubulin acetylation enhances the resistance to paclitaxel-induced cell death. Mechanistically, tubulin acetylation stabilizes the level of the anti-apoptotic protein Mcl-1 by protecting it from degradation [69]. In line with these studies, aberrant expression of tubulin deacetylase HDAC6 has been reported in several cancer cell lines and tumor models [70,71]. In addition, upregulation of HDAC6 increases cell motility in breast cancer cells, thus contributing to cancer metastasis [72], whereas in glioblastoma cells genetic silencing of HDAC6 decreases cellular malignancy and reverses the mesenchymal phenotype [73,74].

**Table 2:** Significance of aberrant expression of tubulin isotypes in cancer.

Tubulin Isotype	Alteration	Cancer Type	Effect	References
TubA1A	High levels	Gastric	Macrophage infiltration in tumor microenvironment	[50]
TubA1B	High level	Hepatocellular carcinoma	Poor overall survival and resistance to paclitaxel	[48]
TubA1C	High level	Glioma	Poor prognosis	[52]
	High level	Lung	Immune cell infiltration	[51]
TubB1	High level	Breast	Taxane resistance	[54]
TubB2	Depletion	Lung	Enhanced sensitivity to Vinca alkaloids	[58]
	Low level	Ovarian and breast	Resistance to taxanes; correlated with advanced stages	[59,60]
	High level	Lung	Biomarker for tumor differentiation and aggressiveness	[61]
Tub3	High level	Ovarian	Correlated with advanced stages	[59]
	High level	Clear cell renal cell carcinoma	Poor prognosis	[56]
	High level	Prostate	Poor overall survival	[65]
	High level	Urinary bladder cancer	Poor prognosis	[57]
	High level	Thyroid carcinoma	Invasive potential and poor prognosis	[62]
TubB4A	High level	Lung	Resistance to paclitaxel	[63]
TubB4B	Depletion	Lung cancer cells	Enhanced sensitivity to Vinca alkaloids	[58]
Tub5	High level	Lung	Biomarker for tumor differentiation and aggressiveness	[61]
	High level	Lung	Treatment response to paclitaxel	[64]

On the other hand, the role of tubulin deacetylase SIRT2 in cancer is controversial, as SIRT2 exerts either tumor-suppressive or oncogenic properties. Overexpression of SIRT2 promotes cell stemness in renal cell carcinoma and endometrial cancer [75,76]. By contrast, overexpression of SIRT2 in lung cancer cell lines induces cell cycle arrest and apoptosis induction [77]. Conversely, the role of ATAT1 in cancer is greatly understudied, as compared with the role of this acetyltransferase in development and non-cancer diseases [27]. Some recent studies have reported that acetylation of tubulin by ATAT1 can affect various pathways, such as cell motility and mitosis, which in turn impinge on cancer cell proliferation, adhesion, invasion, and metastasis [78,79,80]. Notably, despite recent evidence which points to ATAT-1 as a significant player in several types of cancer, little is known about the underlying mechanisms and the relevance of ATAT1 acetyltransferase activity on cancer.

Tubulin detyrosination/tyrosination as well as the associated enzymes have a vital role in several physiological conditions and are associated with malignant transformation and cancer aggressiveness. Tubulin detyrosination seems to represent a selective advantage for cancer cells. Low TTL levels correlate with poor prognoses in several forms of cancer [81,82,83]. For example, tubulin detyrosination is frequent in breast cancer and has been linked to tumor aggressiveness [83]. Concordantly, TTL downregulation induces epithelial–mesenchymal transition and increases in vitro tumor invasion and the in vivo metastatic potential of breast cancer cells [81]. The VASH family, which includes vasohibin-1 (VASH1) and vasohibin-2 (VASH2), is a novel family of angiogenesis regulators [84] that act as tubulin-specific CCPs [40,45]. According to their role in angiogenesis, high expression of VASH1 and VASH2 was associated with poor clinical outcomes in gastric, ovarian, and esophageal squamous carcinoma patients [85,86,87,88]. Despite this, the relationship between the tubulin CCP activity of VASH1/2 and cancer progression or drug sensitivity is still a matter of debate. In ovarian cancer cells, the ablation of VASH2 reduced CCP activity and increased cyclin B1 expression results in increased paclitaxel sensitivity in ovarian cancer cells [89]. 

In a cancer cell context, altered polyglutamylation is linked to tumorigenesis and resistance to drug targeting MTs. A recent study showed that tubulin tyrosine ligase-like 4 (TTLL4) overexpression in breast cancer cells is associated with increased polyglutamylation of TubB, alteration of exosome homeostasis, and brain metastasis [90]. In pancreatic cancer cells, genetic downregulation of TTLL4 attenuates cell proliferation [91]. A new TTLL isoform named TTLL12 has recently been identified [92], and it has been suggested it acts as a potential molecular marker for predicting the invasion and progression of ovarian cancer [93]. On the other hand, the opposite enzyme, AGBL2, promotes tumorigenesis and cancer progression in breast, ovarian, renal, and hepatocellular carcinoma [94,95,96]. This suggests that AGBL2 may serve as a prognostic molecular marker and/or a potential target for therapy. In conclusion, an emerging role of tubulin-modifying enzymes in cancer-associated properties has been identified in different cancer models. Results are summarized in Table 3.

## 3. The Tubulin Code and Its Associated Enzymes in Autophagy

MT dynamic instability and the associated tubulin code have essential roles also in autophagy, a fundamental cellular process. Autophagy is a lysosomal-mediated cellular degradation pathway that exerts a dual role in many cancer types, either supporting cancer growth or acting as a tumor suppressor mechanism (for a review, see [97]). Thus, modulation of autophagy is a promising therapeutic strategy to fight cancer. In this part of the review, we will discuss the impact of tubulin isoforms and tubulin PTMs along with global MT dynamics in the different stages of the autophagic process and the ways in which they modulate the autophagic process in cancer cells. Finally, we will discuss recent findings that have implicated tubulin PTMs and their regulating enzymes in controlling the autophagic process in normal and cancer cells and how tubulin PTMs might be implicated in cancer cell properties or response to therapy.

### 3.1. The Autophagic Machinery: Mechanisms and Regulation

Autophagy is an evolutionarily conserved cellular process in which cellular debris, damaged proteins/organelles, and pathogens are degraded and/or recycled to maintain physiological cell homeostasis. Accordingly, autophagy dysfunction is involved in many diseases, including bacterial or viral infections, neurogenerative diseases, and cancer [97,98]. Beside baseline autophagy, several stimuli can trigger the process, including nutrient deprivation (non-selective macroautophagy) or the intracellular presence of specific degradation targets (selective macroautophagy). In both cases, autophagy originates by the nucleation of a double membrane around the material to be degraded, also referred as phagophore. Membrane expansion and shaping intervenes successively and reflects the form of the engulfed material in selective macroautophagy [99,100]. 

**Figure 1:**
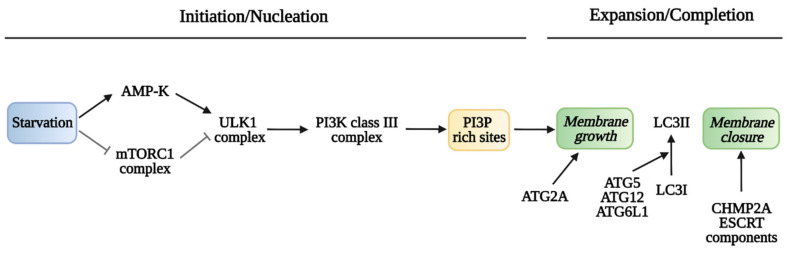
Overview of autophagosome formation. The main complexes involved in initiation/nucleation of the autophagosome membrane and in growth and closure of the autophagosome are reported. Image created with Biorender.

Membrane nucleation is stimulated by the activation of the unc-51-like autophagy activating kinase 1 (ULK1) complex consisting of ULK1, the non-catalytic focal adhesion kinase family interacting protein of 200 KD (FIP200), autophagy-related protein 13 (ATG13) and the ATG101 subunit. The activity of the ULK complex is under multi-layered regulation, so that the ULK1 complex is inhibited by the mammalian target of rapamycin complex 1 (mTORC1) through mTORC1-dependent inactivating phosphorylation of ULK1. Conversely, mTORC1 inactivation by different stimuli promotes ULK1 complex activation and autophagy [101]. In response to amino acid and ATP depletion, a further autophagy-inducing pathway involves the AMP-activated protein kinase (AMPK) that activates the ULK1 complex both by direct phosphorylation of ULK1 and by inhibitory phosphorylation of mTOR. Once activated, the ULK1 complex localizes at sites of condensed cargo via its interaction with the adaptor protein p62, promoting membrane nucleation [102]. Indeed, ULK1-dependent phosphorylation of Beclin 1, a subunit of the class III phosphatidylinositol 3-kinase (PI3K) complex leads to a local increment in phosphatidylinositol 3 phosphate at membrane sites known as pre-autophagosomal structures or omegasomes. Then, membrane expansion is sustained by different membrane sources, especially the endoplasmic reticulum, through the action of the lipid transporter ATG2A [103,104]. The ubiquitin-like ATG5–ATG12 conjugation and the autophagy-related 16-like 1 (ATG16L1) complex are then recruited to phagophores where phosphatidylethanolamine (PtdETn) is conjugated to cytosolic LC3 (known as LC3-I) to produce membrane-associated LC3-II, which acts as a universal adaptor for several protein cargoes and is commonly used as a marker for autophagy activation [105]. Finally, phagophore growth around its cargo leads to membrane convergence and closure of the two membrane ends by the endosomal sorting complexes required for transport (ESCRT) machinery [106]. The different steps in autophagosome biogenesis are outlined in Figure 1. Mature autophagosomes are then transported toward the centrosome, a cell region where lysosomes accumulate [107]. There, autophagosomes and lysosomes fuse to form autolysosomes, where the sequestered materials are degraded by the lysosomal lytic activities. Finally, the degradation products, including sugars, amino acids and nucleic acid precursors, are reused in cellular metabolism [108]. In conclusion, autophagy is a dynamic and complex process that includes autophagosome formation, maturation, fusion with lysosomes, and subsequent degradation of cargoes and autophagosomes themselves into the cytosol. The term “autophagic flux” refers to this whole process and is operationally used to assess whether the process is functional, with autophagosomes forming and dismantling, or blocked in the late stages (autophagic flux blockage), with accumulation of unfused autophagosomes or unfunctional autolysosomes.

### 3.2. The Role of Microtubules in Autophagosome Formation and Fusion with Lysosomes

MT dynamics and MT-based motors have been implicated for a long time in both autophagosome formation and trafficking by studies using drugs interfering with MT assembly and dynamics [109]. 

Starvation-induced autophagosome formation has been shown to require dynamic MTs, since the overall inhibition of their dynamics with the destabilizing drug nocodazole or the stabilizing agent taxol at nanomolar concentrations impairs LC3-II accumulation [110]. Several independent pieces of evidence reinforce the role of MT dynamics in the early stage of autophagosome formation. As suggested by its name, both the nonlipidated and the PtdETn-conjugated forms of LC3 are associated with MTs, either directly or by their interaction with the microtubule-associated protein 1S (MAP1S), a protein that confers cisplatin sensitivity in non-small cell lung cancer cells through autophagy activation [111,112]. Furthermore, several early markers of autophagosome formation (ULK1, Beclin 1, WIPI1, ATG5, ATG12) associated with labile, dynamic MTs suggest that pre-autophagosomal structures originate at dynamic MTs [110,113]. 

**Figure 2:**
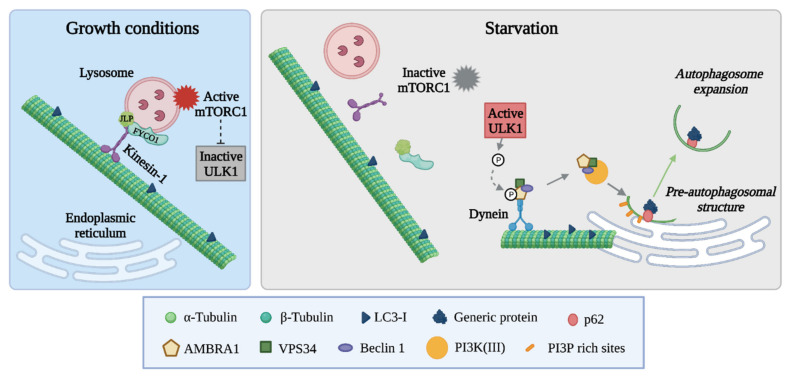
MTs regulate the initiation of autophagy. In normal growth conditions (left panel), lysosomes are maintained at the cell periphery through their FYCO1- and JLP-mediated interaction with the plus-end-directed kinesin 1 and MTs. In these conditions, the lysosome-associated mTORC1 complex is active and inactivates the ULK1 complex. Upon starvation (right panel), lysosomes detach from kinesin 1 and the mTORC1 complex becomes inactive. This stimulates ULK1-dependent phosphorylation of AMBRA1, releasing AMBRA1 from dynein interaction and allowing the translocation of the PI3K complex, comprising Beclin 1/VPS34/AMBRA1, to the endoplasmic reticulum, where it promotes membrane formation at PI3 rich sites. Image created with Biorender.

In addition, MTs regulate two important apical complexes of the autophagic pathway, namely, mTORC1 and PI3K complexes, through MT-associated motor proteins (Figure 2). MTORC1 activity is controlled by lysosome localization, so that in the presence of nutrients the plus end directed kinesin KIF2A and KIF1Bb maintain lysosomes at the cell periphery, keeping lysosome-associated mTORC1 active to suppresses autophagy [114]. Genetic manipulation of the kinesin adaptors FYVE and coiled-coil domain autophagy adaptor 1 (FYCO1) and c-Jun NH2-terminal kinase-associated leucine zipper protein (JLP) has also connected kinesin I activity with lysosomal positioning by showing that kinesin I functional inhibition promotes lysosome translocation to the juxtanuclear area and autophagy activation [114,115]. Starvation-dependent increase in intracellular pH has been implicated in lysosomal centripetal movement through the release of the molecular motors from MTs. In these conditions, dissociation of lysosomes from membrane-associated mTORC1 activators promotes autophagy [114]. These findings, together with recent work on other lysosome-associated nutrient-responsive growth mediators [116,117], have identified MT-dependent lysosome positioning as a dynamic regulator of cell homeostasis [118].

MT-driven intracellular positioning of autophagy mediators is also responsible for the activation of the PI3K complex. Under basal conditions, the autophagy and Beclin 1 regulator 1 (AMBRA1) is sequestered to MTs in a complex with Beclin 1 and phosphatidylinositol 3-kinase (VPS34) through its interaction with the dynein light chain 1 (DLC1), a subunit of the dynein motor complex. Upon autophagy stimulation, ULK1-mediated phosphorylation releases AMBRA1 from dynein interaction, allowing the translocation of Beclin 1/VPS34/AMBRA1 to the omegasome, where the PI3K complex promotes membrane formation [117].

Live cell tracking of individual autophagosomes has shown that autophagosomes form randomly within the cell, then move bidirectionally along stable MTs until they concentrate around the centrosome in the perinuclear region. The centripetal movement of autophagosomes is mediated by the minus-end motor dynein, as shown by using a chemical dynein ATPase inhibitor or by disrupting the dynein complex through p50 dynamitin overexpression [119,120,121]. Autophagosomal centripetal movement is critical for an efficient autophagosome lysosome fusion, since these organelles localize at the perinuclear region. Conversely, plus-end-directed autophagosome movement involves kinesin 1, possibly through the FYCO1 kinesin 1 adaptor that is recruited to autophagosomes by LC3 and Rab7 [110,122,123]. Although the dependence of autophagosomal trafficking on molecular motors has been known from many years, it is still to be elucidated how and why bidirectional movement switches to a net centripetal movement of autophagosomes after autophagy induction and whether the switch in association from labile to stable MTs of nascent vs. mature autophagosomes could contribute to this phenomenon. Furthermore, the role of tubulin PTMs in driving autophagosome formation and trafficking is under debate.

### 3.3. The Tubulin Code in the Autophagic Process

**Figure 3:**
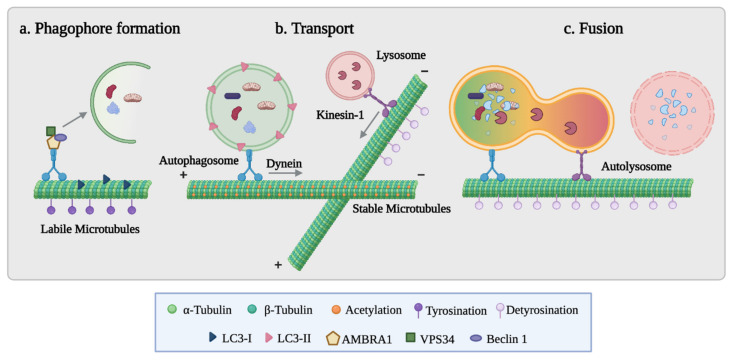
MTs harboring different tubulin post-translational modifications are involved in various stages of autophagy. (**a**) Phagophore formation occurs on labile, dynamic MTs that are characterized by tubulin tyrosination. Upon autophagy stimulation, ULK1-dependent phosphorylation of AMBRA1, which is sequestered on labile MTs in growth conditions, initiates autophagosome formation. (**b**) Closed autophagosomes are transported along stable, acetylated MTs prevalently toward the MT minus end, while lysosomes are enriched on detyrosinated MTs through a kinesin 1-dependent mechanism. (**c**) Autophagosome–lysosome fusion intervenes on detyrosinated MTs. Image created with BioRender.com.

Despite the acquired knowledge on the role of MT dynamics in autophagy, the role of different tubulin isoforms and PTMs in the autophagic process is still not fully clarified. An overview of the current information connecting differently post-translationally modified MTs to the different stages of the autophagic process is presented in Figure 3.

In 2016, a mass spectrometry study reported TubB3 as a binding partner of the autophagic player LC3 [124]. However, the functional significance of this interaction is still unknown and the role of this isoform in autophagy is still undefined. Recently, PCB118, a 2,3′,4,4′,5-pentachlorobiphenyl, has been reported to induce thyroid autophagy by promoting the binding to TubB3 of death-associated protein kinase 2 (DAPK2), a serine/threonine kinase implicated in autophagy and apoptosis, and thereby triggering the DAPK2/PKD/VPS34 pathway [125]. Apart from these studies, little is known of the impact of specific tubulin isoforms on autophagy. 

In the last decade, several groups have focused their attention on the role of tubulin PTMs and their regulating enzymes in controlling the autophagic process (Table 4). Tubulin acetylation is the most studied tubulin PTM in connection with autophagy control. Different studies have used genetic or pharmacological approaches to modulate the enzymes that catalyze the acetylation reaction and have demonstrated the relevance of this PTM in the autophagic process in different experimental models. Upon starvation, tubulin acetylation is involved in regulating the formation of pre-autophagosomal structures and in influencing MT-based autophagosome movements [110]. Indeed, tubulin acetylation determines the spatial localization of autophagosomes and the complete resolving of the autophagic flux [126].

Tubulin acetylation is also essential for the formation of autolysosomes [113,127], and several studies have highlighted the relevance of HDAC6 in autophagosome–lysosome fusion. In cervical carcinoma cells, HDAC6 inhibition promotes tubulin acetylation and impairs serum starvation-induced autophagy by increasing LC3 acetylation and decreasing autophagic flux [128]. Notably, HDAC6 depletion impairs the fusion of autophagosomes and lysosomes by perturbing actin networks [129]. Concordantly, a highly selective HDAC6 inhibitor induces the accumulation of autophagic vacuoles and abrogates the autophagic flux by inhibiting autophagosome–lysosome fusion in glioblastoma cells [130]. In line with these data, HDAC6 has been shown to control autophagic flux in several cancer contexts, including differentiated cancer cells [131,132,133,134] and in cancer stem-like cells [135]. Interestingly, the latter study pointed out that HDAC6 inhibition differentially regulates autophagy in differentiated cancer cells as compared with cancer stem-like cells and identified autophagy as a target for developing anticancer stem cell therapies. The tubulin deacetylase SIRT2 has also been associated with autophagy regulation in several cancer cell lines, although its impact on cancer autophagy is less well understood, in comparison with HDAC6. In colon cancer, SIRT2 downregulation enhanced basal autophagy and mitotic post-slippage death [136]. In human neuroblastoma cells, autophagic flux was inhibited upon SIRT2 overexpression, as evidenced by increased accumulation of LC3-II and p62 proteins. In the same experimental model, SIRT2 overexpression interfered with the accumulation of autophagosomes following proteasome inhibition, leading to neuroblastoma cell death [137]. Notably, a direct link between SIRT2 function in autophagy and modulation of MT acetylation has been already identified in neurodegenerative models, such as Alzheimer’s and Parkinson’s diseases [138].

Less evident is the role of tubulin acetyltransferases in the autophagic process. Dowregulation of ATAT1 acetyltransferase, using a siRNA approach in H1299 lung cancer cells stably expressing the EGFP-LC3 fusion protein, markedly increased EGFP-LC3 puncta compared with control cells, suggesting that ATAT1 may modulate the autophagic process [139]. In another, more recent study, downregulation of ATAT1 promoted tubulin deacetylation and abrogated autophagic flux in response to glucose starvation [126]. Overall, these data support the role of tubulin acetylation as well as the role of deacetylases and acetyltransferases in the control of autophagic flux. Despite this, further work is required to demonstrate the connection between acetylated tubulin-controlled modulation of autophagy and cancer cell features.

**Table 4:** Functional roles of tubulin-modifying enzymes in autophagy progression and cell death.

Enzymes	Cancer Cells	Experimental Approach	Impact on Cancer Autophagy	References
**Lysine Acetyltransferase**				
ATAT1	Lung	Downregulation	Autophagosome accumulation	[139]
**Lysine Deacetylase**				
HDAC6	Glioblastoma, cancer stem-like	Downregulation	Induction of cancer stem cell differentiation by promoting autophagy	[135]
	head and neck	Pharmacological inhibition	Autophagy inhibition	[130]
SIRT2	Neuroblastoma	Overexpression	Inhibition of autophagic flux	[137]
	Colon	Downregulation	Autophagy and mitotic post-slippage death induction	[136]
	Leukemic lines	Pharmacological inhibition	Apoptosis and autophagic cell death	[140]
**Tubulin–tyrosine ligase**				
TTL	Breast, pancreatic	Pharmacological inhibition	Apoptosis and autophagic cell death	[141,142]
**Tubulin-specific carboxypeptidase**				
AGBL2	Hepatocellular carcinoma	Overexpression	Enhanced autophagy by upregulating immunity-related GTPase M	[95]

**Figure 4:**
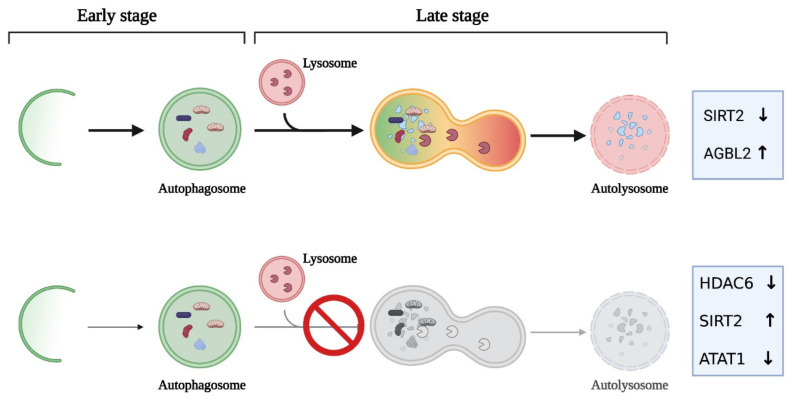
Modulation of tubulin-modifying enzymes impacts differently on autophagic flux. Downregulation of SIRT2 or AGBL2 upregulation promotes functional autophagy. On the contrary, differential expression of the tubulin acetylase ATAT1 or of HDAC6 and SIRT2 deacetylases induces autophagosome accumulation by blocking late autophagic stages. Image created with BioRender.com.

In 2019, Mohan and collaborators showed that tubulin detyrosination/tyrosination has a key role in mediating efficient lysosome–autophagosome encounters during the autophagic process. By using super-resolution microscopy, the authors showed that lysosomes were specifically enriched on detyrosinated MTs and that depletion of detyrosinated MTs reduced the number of autolysosomes, thus highlighting a new role of detyrosinated MTs in key steps of autophagy, namely, autophagosome trafficking and fusion [143]. Concordantly, AGBL2 overexpression increases tubulin detyrosination and enhances autophagy by upregulating immunity-related GTPase M, protecting HCC cells from apoptosis [95]. Collectively, all these studies highlight the role of tubulin PTMs in the regulation of the autophagic process and identify tubulin-modifying enzymes as key modulators of the dynamics of the autophagic flux (Figure 4).

## 4. Autophagy–Microtubule Crosstalk as a Possible Target for Cancer Growth Control

MT-interacting drugs, specifically interacting with either TubA or TubB [7,8,9], are widely used in the therapy of several cancer types. However, limitations on their use due to their neurotoxic side effects and to acquired resistance have stimulated the search for agents with greater effectiveness. In this field, much attention has been given to the capacity of new anti-TubA/TubB agents to affect the autophagic process, their capacity to stimulate or inhibit the autophagic flux and to promote autophagic or apoptosis-related cancer cell death.

Early work showed that the stabilizing drug paclitaxel inhibits autophagosome movement in interphase cells, thereby preventing autophagosome maturation and lysosome fusion in breast cancer cells [144]. The contribution of this block in the autophagic process to paclitaxel-induced cytotoxicity was investigated by the use of the early-stage autophagy inhibitor 3MA. The decreased cell killing effect, observed when autophagosome formation was inhibited by 3MA, demonstrated that blockage of autophagosome traffic and the accumulation of autophagosomes promotes paclitaxel-induced cancer cell death [144]. In line with these findings, several new tubulin binding agents have been shown to activate apoptosis as a result of autophagic flux inhibition in cancer cells [145,146,147,148]. It could be envisaged that induction of autophagosome formation in response to prolonged mitotic arrest and/or mitotic slippage, together with the reduction of autophagosome turnover by flux blockage, could result in the accumulation of autophagosomes, the intracellular persistence of toxic substances and/or damaged organelles, the production of oxygen reactive species, and cytotoxicity [149]. On the other hand, several reports have highlighted a role for autophagy induction in promoting cancer cell survival after exposure to tubulin-binding agents [150,151,152], confirming the dual role of autophagy in cancer [97]. Identifying whether the response to a specific MT-binding agent involves autophagy induction or implies both autophagy induction and flux blockage could be instrumental in determining whether the autophagic response would cooperate or compete with apoptosis in promoting cancer cell death. 

Concerning tubulin PTMs, the direct effects of modulating tubulin PTMs on autophagy induction and cancer growth have not yet been identified. On the other hand, several papers on cancer therapeutics suggest that autophagy can act to promote cell death of tumor cells in response to exposure to HDAC6 inhibitors [130,135,153,154]. Moreover, the novel small molecule SIRT2-specific inhibitor NCO-90/141 has been reported to inhibit cell growth of leukemic cell lines by simultaneously causing apoptosis and autophagic cell death [140]. It should be recognized that the potential mechanisms by which HDAC6 or SIRT2 inhibitors can modulate the autophagic flux and induce autophagic cell death may be only partly dependent on the modulation of tubulin acetylation. It is also possible that deacetylase inhibition can inactivate HSP90 and other chaperones, increasing the levels of denatured proteins and enhancing ER stress signaling [155], thereby influencing autophagy. In line with studies revealing a role for tubulin detyrosination/tyrosination in autophagy, several reports have demonstrated the ability of parthenolide, an inhibitor of MT detyrosination, to induce autophagy and cell death in multiple cancer types, including breast and pancreatic cancer [141,142]. However, in none of these studies the effect of pathenolide on autophagy has been directly linked to its effect on tubulin tyrosination.

Future studies are needed to directly link tubulin PTMs to autophagy regulation. This research will unveil new avenues for modulating autophagy and will offer new perspectives for using tubulin-binding agents and related compounds to target autophagy in cancer.

## 5. Conclusions

MT dynamics have been implicated in a variety of cellular processes that are profoundly deregulated in cancer. Despite this, the contribution of altered MT dynamics to the cancer phenotypes has been often under-investigated. The recent flowering of reports on the role of tubulin PTMs on MT behavior, both on isolated MTs and in cellular contexts, has provided an opportunity to investigate the contribution of tubulin PTMs and tubulin-modifying enzymes in cancer and autophagy. Since autophagy has variable effects on tumor cells, depending on the cellular context and cancer stage, the role of the tubulin code in providing better survival capacity or promoting cancer cell death is an emerging field in cancer studies. As depicted in Figure 4, experimental modulation of the activity of tubulin-modifying enzymes has clearly been shown to impact on the autophagic process. However, both autophagy stimulation, associated with increased survival, and blockage of the autophagic flux, promoting cancer cell death, have been recorded. These findings indicate the need to carefully investigate the molecular determinants that differentiate the two opposing outcomes produced by modulating autophagy. Critical factors, such as cancer progression, cancer metabolic requirements, and autophagy–apoptosis crosstalk, should be carefully examined. Identifying the occurrence of an autophagic block by simple tools, such as late-stage inhibitor co-treatment or GFP-RFP-LC3 expression [105], will enable the determination of whether a genetic or pharmacological intervention on tubulin-modifying enzymes will promote cancer cell death by the cell toxicity associated with autophagosome accumulation [149] and could be usefully applied in the identification of therapeutically promising molecules. 

In conclusion, although the molecular pathways underlying the action of tubulin-modifying enzymes on autophagy need further clarification, the targeted induction of autophagic cell death by inhibitors may represent a new fruitful avenue in cancer therapy.

## Figures and Tables

**Table 1:** Tubulin post-translational modifications (PTMs) and MT dynamics and properties.

Tubulin PTM	Modification Sites	Enzyme	Impact on MTs
Acetylation	TubA Lys40	ATAT1	Resistance to mechanical bending
Deacetylation	TubA Lys40	SIRT2, HDAC6	Sensitivity to mechanical bending
Acetylation	TubB Lys252	SAN acetyltransferase	MT depolymerization
Tyrosination	C-terminal Tyr residue	TTL	Binding of specific MAPs (e.g., MCAK121, CLIP170, dynein/dynactin/BICD2 complex)
Detyrosination	C-terminal Tyr residue	VASH1/2	Associated with MT longevity and binding of specific MAPs (e.g., CENPE, kinesin-2)
Glutamylation/Polyglutamylation	C-terminal Glu residues	Monoglutamylases (TTLL4, -5, 7); Poliglutamylases (TTL-1, -6, -11, -13)	Fine-tuning of MT–MAP interactions
Deglutamylation/Polydeglutamylation	C-terminal Glu residues	CCP -1, -2, -3, -4, -5, -6	Fine-tuning of MT–MAP interactions

**Table 3:** Impact of tubulin-modifying enzymes on different cancer properties.

Enzyme	Cancer Type	Experimental Approach	Impact on Cancer Cell Properties	References
**Lysine AcetylTransferase**				
ATAT1	Lung	Overexpression	Attenuated cell migration, invasion, and metastasis	[79]
	Lung	Overexpression	Drug resistance	[44]
	Lung	Downregulation	Mitotic catastrophe	[80]
	Breast	Downregulation	Attenuated tumor growth	[68]
	Colon	Downregulation	Attenuated cell invasion	[78]
**Lysine Deacetylase**				
HDAC6	Glioblastoma	Downregulation	Proliferation, clonogenicity and cell migration	[73,74]
	Breast	Overexpression	Cell migration	[72]
SIRT2	Lung	Overexpression	Cell cycle arrest and apoptosis induction	[77]
	Endometrial, renal cell carcinoma	Overexpression	Proliferation and stemness	[75,76]
**Tubulin-specific carboxypeptidase**				
VASH2	Ovarian	Downregulation	Drug sensitivity	[89]
AGBL2	Breast, ovarian, prostate and hepatocellular carcinoma	Overexpression	Tumorigenesis and cancer progression	[94,95,96]
**Tubulin monoglutamylase**				
TTLL4	Breast	Overexpression	Increased metastasis	[90]
	Pancreatic	Downregulation	Increased cell proliferation	[91]
